# Myocarditis and Myocardial Diseases—II

**Published:** 1908-08-15

**Authors:** 


### MYOCARDITIS AND MYOCARDIAL DISEASE—II.

### Cloudy Swelling.

Cloudy swelling is a cellular or parenchymatous change, the principal seats of which are the liver, the kidneys, and the heart. It occurs in patients who before death have been the subjects of infective disease with high fever. It is not distinctive of any particular fever, though it is best seen in cases of virulent and fatal rheumatic fever, typhoid fever, diphtheria, and some of the tropical fevers. The change is not produced by the rise of temperature itself, but by the action of toxins. It may occur in acute rheumatism, even when the pyrexia has been abolished by salicylates; and the change is not produced solely by poisons of bacterial origin, for it occurs as one of the earliest effects of poisoning by phosphorus, arsenic, and carbon monoxide.

The change is well characterised by its name, for the affected muscle fibres are larger than normal, their contour is indistinct, and their protoplasm cloudy from the presence of innumerable minute granules of albuminous nature. The nuclei may no longer be visible in stained sections, and the transverse striations are no longer seen. There is no obvious change in the interstitial tissue—no hyperæmia of the blood vessels, and no small round celled exudation. The granules are soluble in acetic acid and in caustic potash, but not in ether. They are not stained black by osmic acid; hence they are not fatty: yet there is no doubt that they are on their way to become fat, for cloudy swelling and fatty changes may be met with side by side, and conditions which, if acute, produce cloudy swelling; if protracted, produce fatty degeneration. According to Cohnheim, Zeigler and others, cells may recover from cloudy swelling if it is not too advanced; this seems likely, upon clinical grounds, but it is only a probability for which there is no definite pathological evidence. To the naked eye the heart is dilated as to all its cavities, but particularly the left ventricle; the walls are consequently thinner than normal. The colour is paler than it should be, and the myocardium lacks both tone and lustre; it has been compared to the appearance that would result if a normal heart were plunged for a few moments into boiling water. The functional power of the heart during life becomes progressively less and less, the rapidity of its beat greater and greater, the area of præcordial dulness increases, the diastole becomes as short as the systole, and the sounds "tic-tac."

### Acute Segmentary Degeneration.

Cloudy swelling may sometimes, especially in virulent rheumatic fever, typhoid fever, diphtheria, and in septic states such as puerperal fever, suppurative pylephlebitis and the like, reach such an intensity that it passes on to a condition of hyaline degeneration of the fibres; the outlines of the latter are obscured, the nuclei no longer stain at all, transverse striation is lost, and the muscle cells look structureless and waxy. Microscopically the heart has a non-lustrous appearance and its cut surface is of a pale grey or yellowish-grey tint.

In dilated hearts whose substance is soft, flabby, and friable, the muscle-cells are often loosened from each other, the individual cells being then easily torn apart or actually separated by transverse clefts. This change has sometimes been called "segmentary myocarditis." The segmentation or dissociation of the fibres is not the manifestation of any definite form of heart disease, but may occur under the most varied condition. It is found, for example, in persons who have died from ischæmic softening or myomalacia (*vide infra*), from certain forms of poisoning, from acute infections, and from nephritis, and even in some cases of death from violence.

It seems quite probable that the dissociation of the muscle-cells takes place partly by reason of the cloudy swelling and hyaline degeneration of the fibres, partly from excessive attempts at contraction, or a perverted mode of contraction; the actual segmentation probably takes place *in articulo mortis*, the degenerative change not leading directly to the separation of the fibres, but only predisposing them to rupture.

### Myomalacia Cordis.

Myomalacia cordis is the name given to softening of the cardiac muscle consequent on arterial anæmia or ischæmia, the commonest causes of which are sclerosis, atheroma, and calcification, and thrombosis of the coronary arteries or their branches. More rarely it may be due to embolism of these arteries. There may be no symptoms produced by the lesion if it comes on gradually and is small in extent; the accumulation of small patches here and there, together with the secondary changes which occur in them, ultimately lead to the production of a fibroid heart in a way that will be described below. If a large area of the heart becomes affected by myomalacia, the chief symptoms will be those of a sudden "cardiac seizure," with angina. Sudden death will result if the lesion is a sudden obstruction of a main coronary artery.

The appearance of the areas of softening differs according to their age and the amount of blood contained in them. Shortly after the occurrence of the ischæmia the patches are still firm, and appear only as dull yellowish discolourations of the heart muscle. They cannot be distinguished from fatty or fibroid patches until examined microscopically. After a time they become softened and friable, and assume a yellowish-white tint. Sometimes, if the substance has already softened, the cut surface of a cross-section sinks in so as to become concave.

If in consequence of the obliteration or occlusion of the arterioles an extravastation of blood takes place from the capillaries, a hæmorrhagic infarct is produced. The infarcted area is at first either uniformly dark red or mottled with dark red, brown, and yellow. It may be yellow in the middle and red at the border. After a time it may become greyish-yellow, greyish-brown, or even of a rusty tint. Later on both the anæmic and the hæmorrhagic areas take on a greyish translucent appearance, and the surface retracts when cut.

